# Alteration in tumoural PD-L1 expression and stromal CD8-positive tumour-infiltrating lymphocytes after concurrent chemo-radiotherapy for non-small cell lung cancer

**DOI:** 10.1038/s41416-019-0541-3

**Published:** 2019-08-07

**Authors:** Kazue Yoneda, Taiji Kuwata, Masatoshi Kanayama, Masataka Mori, Toshinori Kawanami, Kazuhiro Yatera, Takayuki Ohguri, Masanori Hisaoka, Toshiyuki Nakayama, Fumihiro Tanaka

**Affiliations:** 1 Second Department of Surgery (Chest Surgery), University of Occupational and Environmental Health Japan, Kitakyushu, Japan; 2 Department of Respiratory Medicine, University of Occupational and Environmental Health Japan, Kitakyushu, Japan; 3 Department of Radiology, University of Occupational and Environmental Health Japan, Kitakyushu, Japan; 4 Department of Pathology and Oncology, University of Occupational and Environmental Health Japan, Kitakyushu, Japan; 5 Department of Pathology, University of Occupational and Environmental Health Japan, Kitakyushu, Japan

**Keywords:** Non-small-cell lung cancer, Tumour biomarkers

## Abstract

**Background:**

Consolidation treatment with an anti-PD-L1 antibody, durvalumab, following concurrent chemo-radiotherapy (cCRT) has become a new standard of care for locally advanced non-small cell lung cancer (NSCLC). The rationale of PD-L1 blockade after cCRT is based on preclinical evidence suggesting that chemotherapy and radiotherapy up-regulate tumoural PD-L1 expression, which has not been shown in clinical studies.

**Methods:**

To examine alteration in tumoural PD-L1 expression (tumour proportion score, TPS) and density of stromal CD8-positive tumour-infiltrating lymphocytes (CD8 + TILs) after cCRT, paired NSCLC samples obtained before and after cCRT were reviewed in comparison with those obtained before and after drug therapy.

**Results:**

PD-L1 expression was significantly up-regulated after cCRT (median TPS, 1.0 at baseline versus 48.0 after cCRT; *P* < 0.001), but not after drug therapy. There was no significant correlation between baseline TPS and post-cCRT TPS. CD8 + TIL density was significantly increased after cCRT (median, 10.6 versus 39.1; *P* < 0.001), and higher post-cCRT CD8 + TIL density was associated with a higher pathologic response and with a favourable survival (*P* = 0.019).

**Conclusion:**

Tumoural PD-L1 expression was up-regulated after cCRT, which provides pathologic rationale for PD-L1 blockade following cCRT to improve prognosis. Stromal CD8 + TIL density was also increased after cCRT, and higher post-cCRT CD8 + TIL density was a favourable prognostic indicator.

## Background

Non-small cell lung cancer (NSCLC) accounts for ~85% of lung cancer, which is the leading cause of cancer deaths worldwide.^1^ More than 30% of NSCLC patients present with locally advanced and unresectable disease, and concurrent chemo-radiotherapy (cCRT) has been employed as a standard treatment of care for such patients. However, the prognosis achieved with cCRT remains un-satisfactory with the 5-year overall survival (OS) rate of 15%.^2^ Recently, a randomised controlled trial demonstrated that the consolidation treatment with durvalumab, an antibody against programmed death ligand-1 (PD-L1), provided a significant survival benefit among patients who did not have disease progression after cCRT.^3,4^ Based on the results, durvalumab treatment following cCRT has become a new standard of care for locally advanced and unresectable NSCLC patients with good performance status (PS).^5^

The rationale of addition of PD-L1 blockade following cCRT is based on preclinical evidence suggesting that chemotherapy^6^ and radiotherapy^7,8^ up-regulate PD-L1 expression on tumour cells. Up-regulation of tumoural PD-L1 leads to acquired resistance to radiotherapy, which may be overcome by PD-L1 blockade.^7,8^ However, there has been reported no clinical evidence showing up-regulation of tumoural PD-L1 expression after cCRT. Only one clinical study to examine alteration in PD-L1 expression by using paired NSCLC samples obtained before and after cCRT showed a significant decrease in PD-L1 expression after cCRT,^9^ which is contrary to preclinical results.^7,8^ In the present study, to assess the impact of cCRT on tumour immune microenvironment in NSCLC, we examined alteration in tumoural PD-L1 expression and in density of stromal CD8-positive tumour-infiltrating lymphocytes (CD8 + TILs) in paired histologic samples that had been obtained before and after cCRT in comparison with that in paired specimens obtained before and after drug therapy.

## Methods

### Patients

NSCLC patients who received pre-operative treatment followed by surgery from January 2008 through December 2017 at our institute were retrospectively reviewed. Patients were eligible when paired tumour specimens obtained before and after preoperative treatment were available for pathological evaluation and for immunohistochemistry (IHC) to examine tumoural PD-L1 expression and stromal CD8 + TILs. No patients received radiotherapy alone. Seven patients who achieved pathologic complete response with preoperative treatment were excluded, because tumoural PD-L1 expression could not be evaluable. A total of 23 patients who underwent cCRT were included and were reviewed in comparison with 18 patients who underwent drug therapy alone as preoperative treatment (Supplementary Fig. 1).

Clinical stage (c-stage) was determined according to the current tumour, node, metastases (TNM) classification.^10^ Pathological response to preoperative treatment was evaluated according to the ‘General rule for clinical and pathological record of lung cancer (7th edition) by the Japan Lung Cancer Society’ as follows: therapeutic effect 0, no therapeutic effect; 1, residual viable cancer cells detected in ‘≥1/3’ of resected tumour; 2, residual viable cancer cells detected in ‘<1/3’ of resected tumour; 3, no residual viable cancer cells.^11^

### Evaluation tumour immune microenvironment

PD-L1 expression on tumour cells and density of stromal CD8 + TILs were evaluated with IHC.^9,12–14^ Serial 4-µm sections were cut from each formalin-fixed and paraffin-embedded tumour specimen. For evaluation of PD-L1 expression, sections were heated in 1 mmol/L ethylenediaminetetraacetic acid (pH 8.0) at 98°C for 15 min for antigen retrieval, and were incubated in 3% hydrogen peroxide for 10 min to inactivate endogenous peroxidase. After blocking with Protein Block Serum-Free (Agilent Technologies, Carpinteria, CA) for 30 min, sections were incubated with a rabbit anti-PD-L1 monoclonal antibody (clone E1L3N,^12,13^ Cell Signaling Technology Japan, Tokyo, Japan) diluted at 1:200 for 1 h at room temperature. Sections were then washed and incubated with SignalStain Boost IHC Detection Reagent HRP Rabbit (Cell Signaling Technology Japan) for 30 min. Thereafter, they were visualised with DAB + Liquid (Agilent Technologies) and counterstained with haematoxylin. Paraffin-embedded cell pellets (KARPAS-299 as PD-L1-positive cells and PC-3 as PD-L1 negative cells, Cell Signaling Technology Japan) served as controls for PD-L1 immuno-staining. For evaluation of CD8-positive lymphocytes, slide was stained with an anti-CD8 antibody (clone C8/144B; Aglient) using the DAKO Autostainer Link 48 (Agilent Technologies) according to the manufacturer’s protocol.

Each slide was independently evaluated by two of the investigators (K.Y. and F.T.) without the knowledge of any clinical data. The percentage of tumour cells with membrane staining for PD-L1 was recorded and represented as tumour proportion score (TPS). A minimum of 100 tumour cells were evaluated to calculate TPS. The percentage of CD8-positive lymphocytes among total nucleated cells in the stromal compartments were defined as the CD8 + TIL density.^9,14^ When a discrepancy was found between the two investigators, the slide was reviewed via their simultaneous examination using a double-headed microscope to achieve a consensus.

### Statistical analysis

Proportions of categorical data were compared by the chi-square test or the Fisher’s exact test, as appropriate. Continuous data were compared with a non-parametric test (Wilcoxon signed rank test for paired data or Mann–Whitney U-test for un-paired data). Spearman’s rank correlation coefficients (two-sided) were used to evaluate correlations between two parameters. Receiver operating characteristic (ROC) curve analyses were performed to determine the optimal cut-off value of TPS for PD-L1 expression and CD8 + TIL density.

A telephone inquiry was made if the patient did not come to our clinic for a routine follow-up. The Kaplan–Meier method was used to estimate probability of recurrence-free survival (RFS) and OS, and survival differences were analysed by the log-rank test. The hazard ratio (HR) and 95% confidence interval (CI) were calculated for each variable.

Differences were considered to be statistically significant for *P-*values < 0.05. All statistical analyses were performed with the SPSS version 21 software (IBM, Armonk, NY).

## Results

### Patient characteristics

Among 23 patients who received cCRT, the majority of patients had c-stage III disease. All patients received platinum-based chemotherapy in combination with concurrent radiotherapy with the median dose of 60 Gy followed by complete resection. Seventeen (73.9%) of 23 patients achieved therapeutic effect 2 with cCRT. In the drug-therapy group, only five (27.8%) of 18 patients achieved therapeutic effect 2 with pre-operative treatment (Table 1).

**Table 1:** Characteristics of patients

Characteristic		Drug-therapy group (*n* = 18)	Concurrent chemo-radiotherapy group (*n* = 23)	*P*
Age, median (years)		68.0 (46–84)	62.0 (40–73)	0.045
Sex	Female	5 (27.8%)	5 (21.7%)	0.724
	Male	13 (72.2%)	18 (78.3%)	
Smoking status	Never	5 (27.8%)	4 (17.4%)	0.471
	Former or current	13 (72.2%)	19 (82.6%)	
Brinkman index, median (pack-year)		47 (0–90)	40 (0–88)	0.895
Histology	Adenocarcinoma	10 (55.6%)	14 (60.9%)	0.760
	Squamous cell carcinoma	7 (38.9%)	7 (30.4%)	
	Others	1 (5.6%)	2 (8.7%)	
Driver mutation	Present	7 (46.7%)	5 (29.4%)	0.467
	(*EGFR/ALK*)	(5/2)	(4/1)	
Clinical stage	II	2 (11.1%)	5 (21.7%)	0.438
	(IIA/IIB)	(1/1)	(0/5)	
	III	16 (88.9%)	18 (78.3%)	
	(IIIA/IIIB/IIIC)	(9/3/4)	(11/6/1)	
Radiotherapy dose (Gray)		–	60 (30–70)	
Drug therapy	Platinum doublet	7 (38.9%)	23 (100.0%)	<0.001
	Other cytotoxic regimen	1 (5.6%)	0	
	Cisplatin (BAI)	4 (22.2%)	0	
	TKI	6 (33.3%)	0	
	(Gef/Erlo/Alec)	(4/1/1)		
Pathologic response	Therapeutic effect 1	13 (72.2%)	6 (26.1%)	0.005
	Therapeutic effect 2	5 (27.8%)	17 (73.9%)	

Data represented as absolute counts (%) or median (range)

*EGFR* epidermal growth factor receptor gene, *ALK* anaplastic large-cell lymphoma kinase gene, *BAI* bronchial arterial infusion, *TKI* tyrosine kinase inhibitor, *Gef* gefitinib, *Erlo* erlotinib, *Alec* alectinib

Therapeutic effect 1, residual viable cancer cells detected in ‘≥1/3’ of resected tumour

Therapeutic effect 2, residual viable cancer cells detected in ‘<1/3’ of resected tumour

### Alteration in PD-L1 expression on tumour cells

**Fig. 1:**
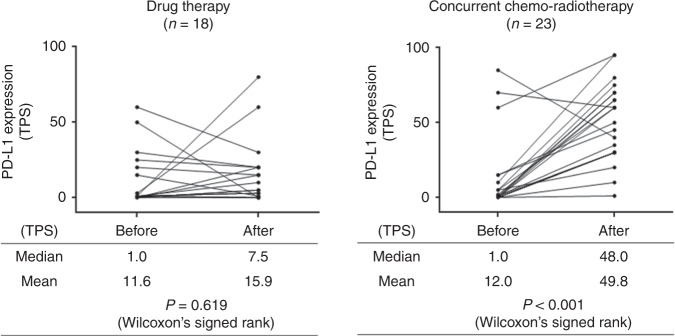
Alteration in PD-L1 expression on tumour cells after drug therapy or concurrent chemo-radiotherapy. PD-L1 programmed death ligand-1, TPS tumour proportion score, CT chemotherapy, cCRT concurrent chemo-radiotherapy

After cCRT, 21 patients (91.3%) showed an increase in TPS, and the other two patients showed a decrease. After drug therapy, 10 patients showed an increase in TPS, whereas the other eight patients showed a decrease. TPS was significantly increased after cCRT (median TPS, 1.0 at the baseline versus 48.0 after cCRT; *P* < 0.001) but was not after drug therapy (Fig. 1). The post-treatment TPS was significantly higher in the cCRT group than in the drug-therapy group (median TPS, 48.0 and 7.5, respectively; *P* < 0.001), whereas baseline TPS was similar (median TPS, 1.0 in both group; *P* = 0.959). There was no significant correlation between baseline TPS of and post-treatment TPS in the cCRT group or in the drug-therapy group (Supplementary Fig. 2A).

In the cCRT group, PD-L1 expression was significantly increased after treatment not only in patients with positive PD-L1 expression at the baseline (baseline TPS, ≥1) but also in patients with negative PD-L1 expression at the baseline (baseline TPS, 0) (Supplementary Fig. 2B). There was no significant difference in the post-cCRT TPS between patients with negative baseline PD-L1 expression and those with positive baseline PD-L1 expression (median post-cCRT TPS, 30.0 versus 60.0; *P* = 0.117). PD-L1 expression was significantly increased after cCRT regardless of pathologic response (Supplementary Fig. 2C). There was no significant difference in baseline or post-cCRT TPS according to pathologic response (median baseline TPS, 0.0 in therapeutic effect 1 group and 1.0 for therapeutic effect 2 group *P* = 0.081; median post-cCRT TPS, 70.0 and 40.0, respectively; *P* = 0.052).

### Alteration in stromal CD8 + TIL density

**Fig. 2:**
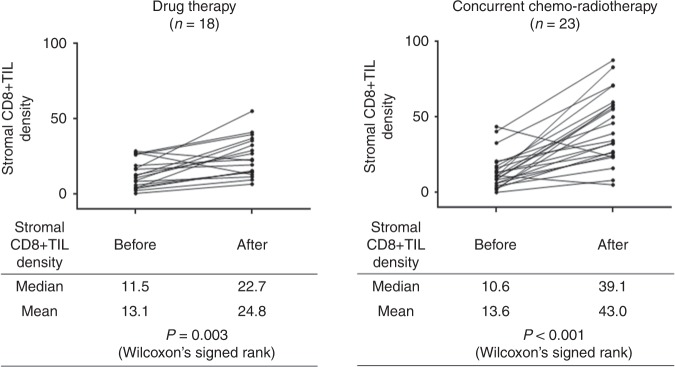
Alteration in density of CD8-positive tumour-infiltrating lymphocytes after drug therapy or concurrent chemo-radiotherapy. CD8 + TIL CD8-positive tumour-infiltrating lymphocyte, CT chemotherapy, cCRT concurrent chemo-radiotherapy

After cCRT, 22 patients (95.7%) showed an increase in CD8 + TIL density, and only one patient showed a decrease. After drug therapy, 16 patients showed an increase in CD8 + TIL density, and only two patients showed a decrease. CD8 + TIL density was significantly increased after cCRT (median density, 10.6 at the baseline versus 39.1 after cCRT; *P* < 0.001) and was also increased after drug therapy (11.5 at the baseline versus 22.7 after drug therapy; *P* = 0.003) (Fig. 2). The post-treatment CD8 + TIL density was significantly higher in the CRT group than in the drug-therapy group (median, 39.1 and 22.7; *P* = 0.001), whereas baseline CD8 + TIL density was similar in both groups (median, 10.6 and 11.5, respectively; *P* = 0.874). There was no significant correlation between baseline CD8 + TIL density and post-treatment CD8 + TIL density in the cCRT group or in the drug-therapy group (Supplementary Fig. 3A).

In the cCRT group, CD8 + TIL density was significantly increased after treatment regardless of pathologic response (Supplementary Fig. 3B). However, post-cCRT CD8 + TIL density was significantly higher in therapeutic effect 2 group than in therapeutic effect 1 group (*P* = 0.001), whereas no significant difference in baseline CD8 + TIL density between two groups was observed (*P* = 0.309).

### Prognosis

**Fig. 3:**
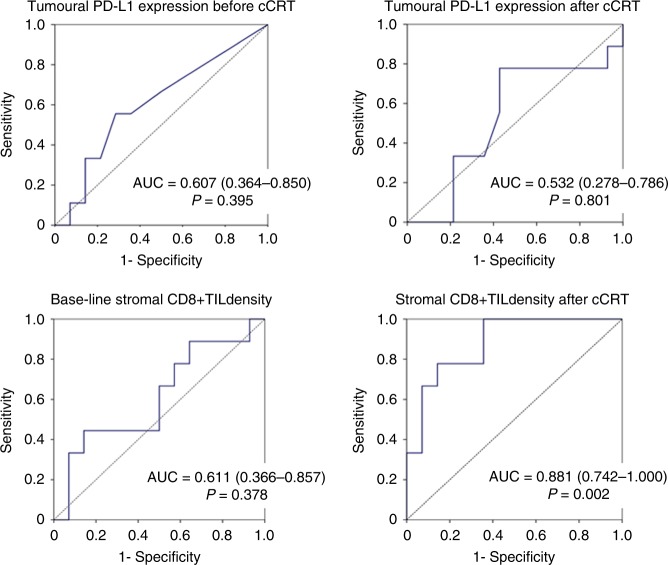
Receiver operating characteristics curve for programmed death ligand-1 (PD-L1) expression on tumour cells or density of stromal CD8-positive tumour-infiltrating lymphocytes to predict death or recurrence after surgery following concurrent chemo-radiotherapy. PD-L1 programmed death ligand-1, TPS tumour proportion score, cCRT concurrent chemo-radiotherapy, CD8 + TIL CD8-positive tumour-infiltrating lymphocyte, AUC area under curve

Prognostic implication of tumoural PD-L1 expression status and stromal CD8 + TIL density was assessed in 23 patients who underwent cCRT followed by surgery. ROC curve analyses revealed that only the post-cCRT CD8 + TIL density provided a significant prognostic value to predict death or tumour recurrence with the optimal cut-off value of 40 (Fig. 3). The change in TPS or in CD8 + TIL density after cCRT provided no significant prognostic value (*P* = 1.000 for change in TPS, *P* = 0.488 for change in CD8 + TIL density).

**Fig. 4:**
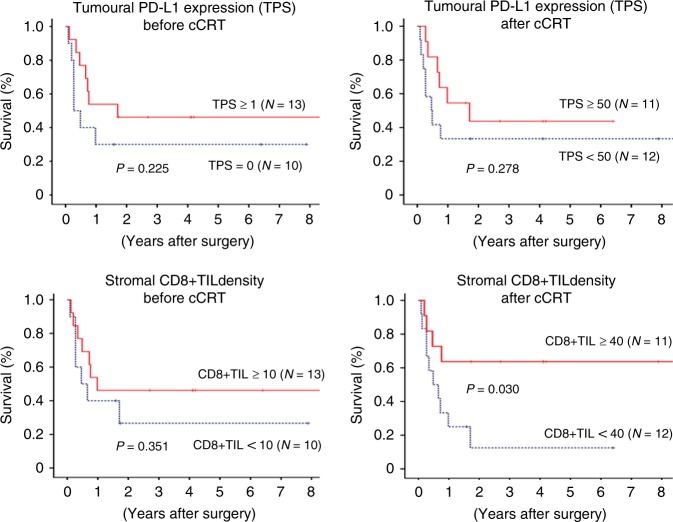
Recurrence-free survival curve after surgery following concurrent chemo-radiotherapy according to programmed death ligand-1 (PD-L1) expression on tumour cells or density of CD8-positive tumour-infiltrating lymphocytes. PD-L1 programmed death ligand-1, TPS tumour proportion score, cCRT concurrent chemo-radiotherapy, CD8 + TIL CD8-positive tumour-infiltrating lymphocyte

**Fig. 5:**
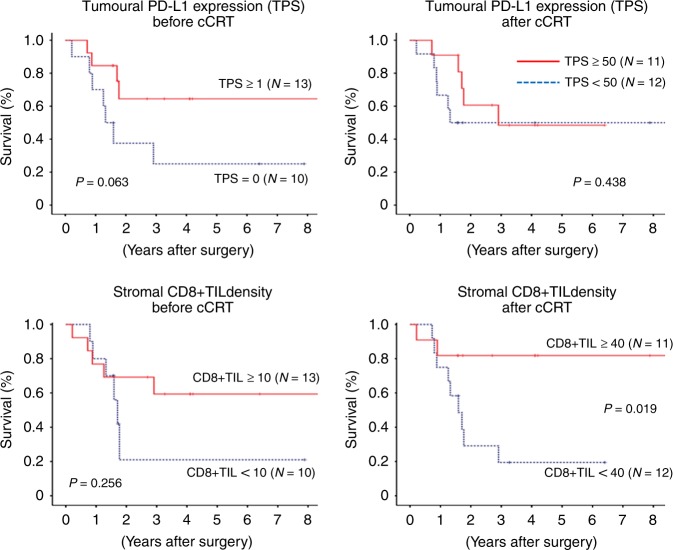
Overall survival curve after surgery following concurrent chemo-radiotherapy according to programmed death ligand-1 (PD-L1) expression on tumour cells or density of stromal CD8-positive tumour-infiltrating lymphocytes. PD-L1 programmed death ligand-1, TPS tumour proportion score, cCRT concurrent chemo-radiotherapy, CD8 + TIL CD8-positive tumour-infiltrating lymphocyte

Patients with higher post-cCRT CD8 + TIL density (≥40) showed significantly favourable RFS and OS than those with lower post-cCRT CD8 + TIL density (Figs. 4 and 5). For other parameters, RFS and OS were analysed using the median value (1 for baseline TPS, 50 for post-cCRT TPS and 10 for baseline CD8 + TIL density, respectively), which provided no significant prognostic value (Figs. 4 and 5).

## Discussion

The present study first demonstrated a significant up-regulation of PD-L1 expression on tumour cells after cCRT in paired clinical samples, which was documented regardless of PD-L1 expression status before cCRT. In addition, the stromal CD8 + TIL density was also significantly increased after cCRT, which was correlated with a higher pathologic response and with a favourable prognosis.

CD8 is a transmembrane glycoprotein that serves as a co-receptor for the T cell receptor (TCR). CD8 is predominantly expressed on cytotoxic T cells (CTLs) that play central roles in cell-mediated immune attack, whereas it can be expressed on natural killer and dendritic cells.^15–17^ Accordingly, CD8 is generally recognised as a marker of CTLs.　A number of clinical studies revealed that a higher density of CD8 + TILs was associated with a favourable prognosis in a variety of malignant tumours including NSCLC,^17–22^ The present study also showed that a higher stromal CD8 + TIL density after cCRT was associated with a favourable prognosis (Figs. 3–5). In the present study, we first demonstrated a significant increase in CD8 + TIL density after cCRT (Fig. 2). In addition, of noted, the baseline CD8 + TIL density was not correlated with the post-cCRT CD8 + TIL density (Supplementary Fig. 3A), or provided no significant prognostic value (Figs. 3–5). Accordingly, before initiation of cCRT, it may be difficult to predict CD8 + TIL density after cCRT, which is a significant prognostic marker.

In the present study, higher stromal CD8 + TIL density after cCRT was associated with a higher pathologic response (therapeutic effect 2), which is reasonably explained by experimental results showing that CTLs play a crucial role in anti-tumour effects of radiation.^23,24^ It has been generally considered that the main mechanism of killing tumour cells by ionising radiation is the direct DNA damage. However, several preclinical studies have revealed that radiation therapy may induce anti-tumour immune response through promoting release of neo-antigen from tumour cells.^23,25–27^ For example, Takeshima and co-workers showed that tumour-inhibitory effect of radiation was almost completely abolished in tumour-bearing mice when CD8-positive T cells were depleted.^23^ These preclinical results suggest that the immune attack by CD8 + TILs is an important mechanism of anti-tumour effect of radiation therapy. Therefore, when irradiated, tumour cells may evade from immune attack by CD8 + TILs through several mechanisms, which leads to resistance to radiotherapy. The PD-1/PD-L1 axis is the most important mechanism responsible for immune evasion of tumour cells.^28^ PD-L1 expressed on tumour cells binds to PD-L1 on CTLs, which results in down-regulation of immune activity of CTLs. PD-L1 on tumour cells may be up-regulated in response to immune attack induced by radiotherapy, which has been demonstrated in preclinical models.^7,8^ The present study is the first clinical study showing up-regulation of PD-L1 after cCRT (Fig. 1). PD-L1 expression may be drastically changed during clinical course especially through a variety of treatment such as radiotherapy and chemotherapy.^29^ In the present study, pre-treatment PD-L1 status was not correlated with post-treatment PD-L1 status, indicating that post-treatment PD-L1 expression cannot be predicted by pre-treatment PD-L1 status. These results may provide a rationale of PD-L1 blockade following cCRT regardless of pre-treatment PD-L1 status. In fact, consolidation durvalumab treatment following cCRT significantly prolonged progression-free survival and OS in locally advanced NSCLC regardless of baseline PD-L1 status (either for TPS < 25 or for TPS ≥ 25).^3,4^ In the present study, the majority of patients showed an increase in TPS after cCRT (Fig. 1). Accordingly, we could not compare clinical and pathological characteristics between patients with increased TPS and those with decreased TPS to reveal predictive factors of up-regulation of TPS after cCRT, which should be examined in a large-scale study.

The present study has several limitations due to a variety of weakness. First, this study was a retrospective single-institutional study of a small number of patients. We employed ROC curve analyses to determine the optimal cut-off values of TPS for PD-L1 expression and stromal CD8 + TIL density in prediction of death or tumour recurrence, which may not be suitable for the present study with a small number of patients. The optimal cut-off values should be examined in a large-scale study. Second, the PD-L1 IHC assay employed in the present study is not a companion or complementary diagnostic assay approved for clinical use but is a laboratory-developed test using an antibody against PD-L1 (clone E1L3N). A prospective multi-institutional study revealed that the E1L3N assay showed similar results to trial-validated PD-L1 IHC assays (28–8 and 22C3 assays).^12^ However, results in the present study shall be validated in a prospective multi-institutional study with a validated IHC assay. Third, there are vast heterogeneity in patient characteristics such as radiation dose and chemotherapy regimen employed in cCRT. The conflicting results between the present study showing increased PD-L1 expression after cCRT and in the previous study showing decreased PD-L1 expression^9^ may be caused by differences in several patient characteristics. Tumoural PD-L1 status is employed in routine clinical practice as a predictive marker in blockade of PD-1/PD-L1 axis for advanced NSCLC.^29–31^ However, the prognostic value of PD-L1 status remains controversial, and some studies indicated a poor prognosis in patients with strong PD-L1 expression but others indicated a favourable prognosis.^13,29,32,33^ In the present study, pre-treatment or post-treatment PD-L1 status provided no significant prognostic value in patients who underwent cCRT followed by surgery, whereas high TPS seemed to be associated with a favourable OS (Fig. 5). The prognostic implication should be also assessed and validated in a future prospective study. In addition, 18 patients in the drug-therapy group were treated with a wide variety of regimens including tyrosine kinase inhibitors (TKIs) prescribed for six patients. Cytotoxic agents and TKIs show anti-tumour activity through different mechanism of action and may potentially cause different immune responses. Due to the small number of eligible patients in the study, we did not compare tumour microenvironment between patients treated with cytotoxic agents and those treated with TKIs in the present study, which will be examined in a future study. Finally, the present study provided no data on clinical response to anti-PD-L1 treatment following cCRT. A future study to investigate the effect of post-cCRT tumoural PD-L1 expression status and stromal CD8 + TIL density on anti-PD-L1 treatment following CRT should be conducted.

In conclusion, PD-L1 expression on tumour cells and stromal CD8 + TIL density were significantly increased after cCRT. There was no significant correlation between pre-treatment and post-treatment tumoural PD-L1 or stromal CD8 + TIL density. The higher CD8 + TIL density after cCRT was associated with a higher pathologic response and with a favourable survival.

## Supplementary information

Supplementary Infirmation

## Data Availability

Requests for data and reagents can be made by contacting the corresponding author.
