# Supplementary material for: Alteration in tumoural PD-L1 expression and stromal CD8-positive tumour-infiltrating lymphocytes after concurrent chemo-radiotherapy for non-small cell lung cancer
Source: Br J Cancer. 2019 Aug 7;121(6):490–6. doi: 10.1038/s41416-019-0541-3 (PMC6738061; doi:10.1038/s41416-019-0541-3)
Supplement: Supplementary file 1 — Supplementary Infirmation [file 41416_2019_541_MOESM1_ESM.pptx]

## Slide 1
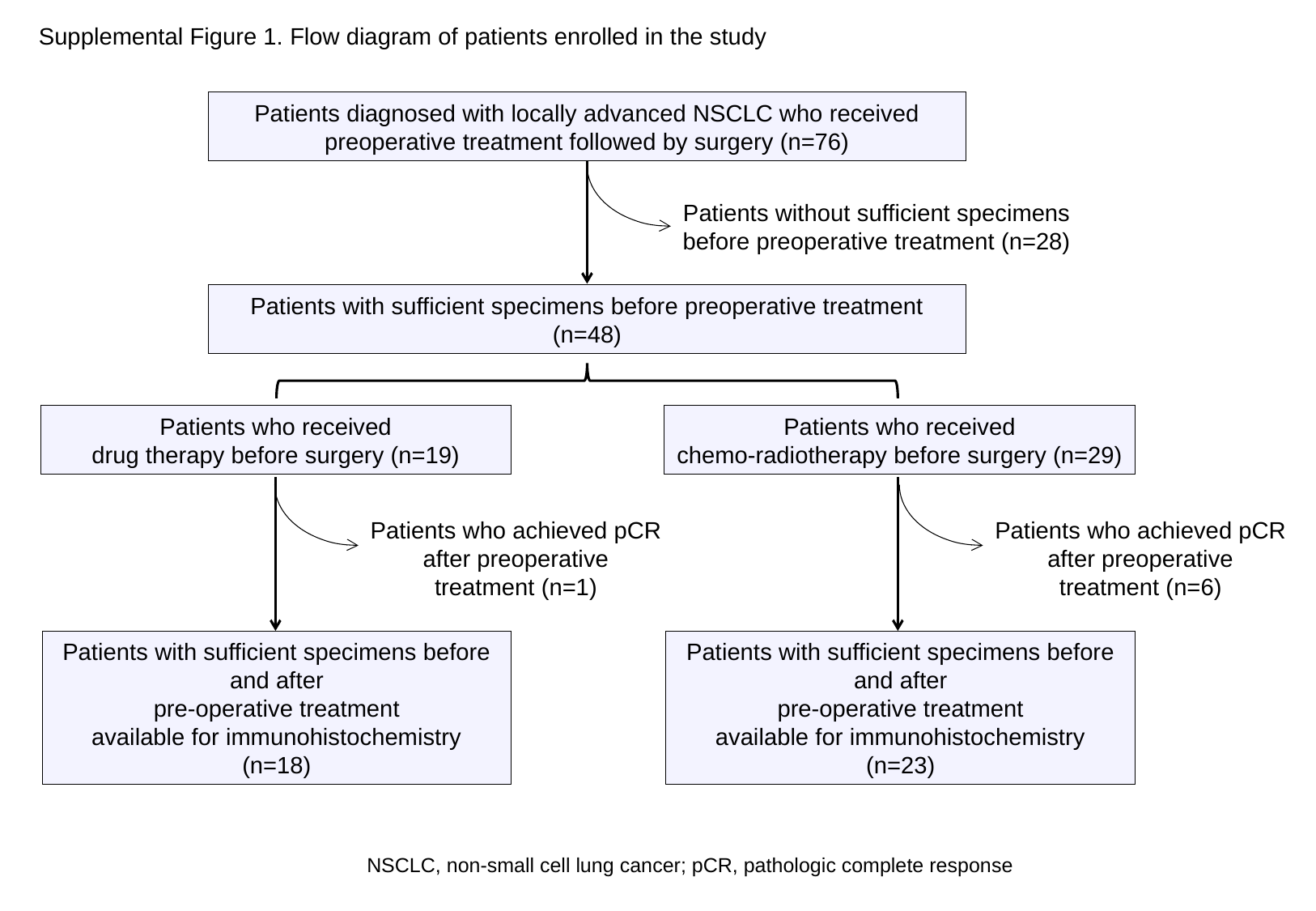

Supplemental Figure 1. Flow diagram of patients enrolled in the study
Patients diagnosed with locally advanced NSCLC who received
preoperative treatment followed by surgery (n=76)
Patients without sufficient specimens
before preoperative treatment (n=28)
Patients with sufficient specimens before preoperative treatment (n=48)
Patients who received
drug therapy before surgery (n=19)
Patients who received
chemo-radiotherapy before surgery (n=29)
Patients who achieved pCR after preoperative treatment (n=1)
Patients who achieved pCR after preoperative treatment (n=6)
Patients with sufficient specimens before and after
pre-operative treatment
available for immunohistochemistry
(n=23)
Patients with sufficient specimens before and after
pre-operative treatment
available for immunohistochemistry
(n=18)
NSCLC, non-small cell lung cancer; pCR, pathologic complete response

## Slide 2
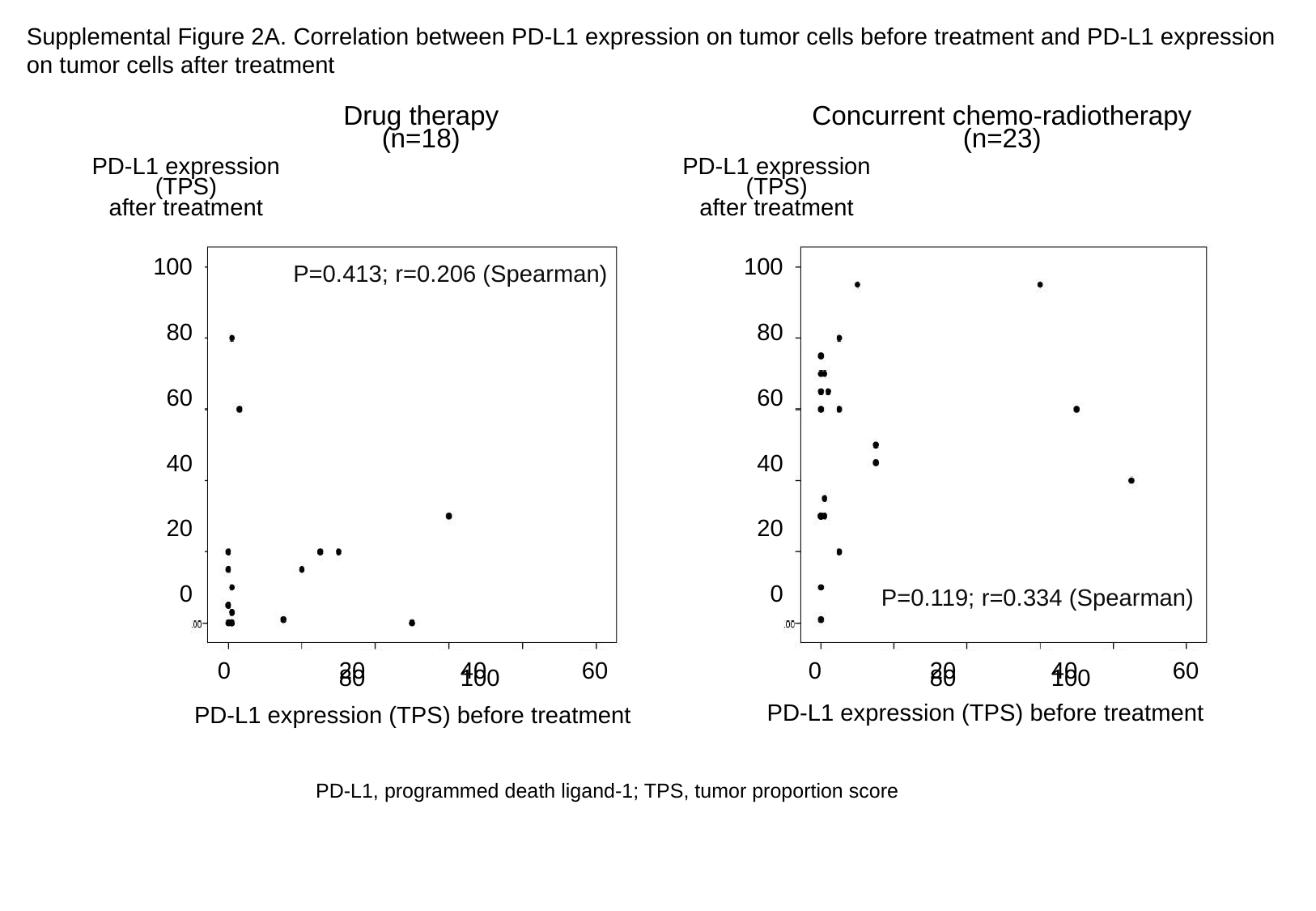

Supplemental Figure 2A. Correlation between PD-L1 expression on tumor cells before treatment and PD-L1 expression on tumor cells after treatment
Drug therapy
(n=18)
Concurrent chemo-radiotherapy
(n=23)
PD-L1 expression
(TPS)
after treatment
100
80
60
40
20
0
0	20	40	60	80	100
PD-L1 expression (TPS) before treatment
PD-L1 expression
(TPS)
after treatment
100
80
60
40
20
0
0	20	40	60	80	100
PD-L1 expression (TPS) before treatment
P=0.413; r=0.206 (Spearman)
P=0.119; r=0.334 (Spearman)
PD-L1, programmed death ligand-1; TPS, tumor proportion score

## Slide 3
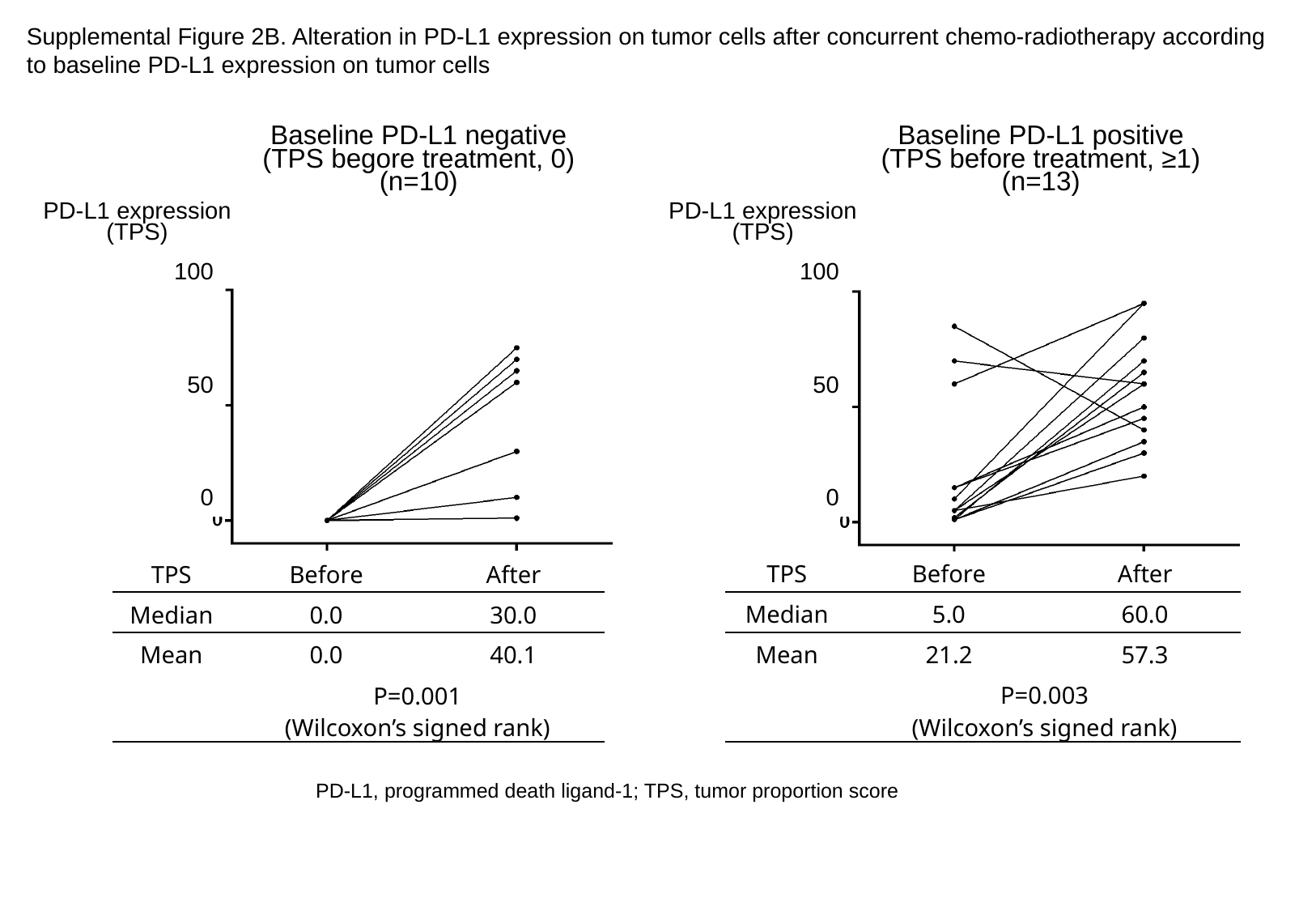

Supplemental Figure 2B. Alteration in PD-L1 expression on tumor cells after concurrent chemo-radiotherapy according to baseline PD-L1 expression on tumor cells
Baseline PD-L1 negative
(TPS begore treatment, 0)
(n=10)
Baseline PD-L1 positive
(TPS before treatment, ≥1)
(n=13)
PD-L1 expression
(TPS)
100
50
0
PD-L1 expression
(TPS)
100
50
0
| TPS | Before | After |
| --- | --- | --- |
| Median | 5.0 | 60.0 |
| Mean | 21.2 | 57.3 |
| | P=0.003 (Wilcoxon’s signed rank) | |
| TPS | Before | After |
| --- | --- | --- |
| Median | 0.0 | 30.0 |
| Mean | 0.0 | 40.1 |
| | P=0.001 (Wilcoxon’s signed rank) | |
PD-L1, programmed death ligand-1; TPS, tumor proportion score

## Slide 4
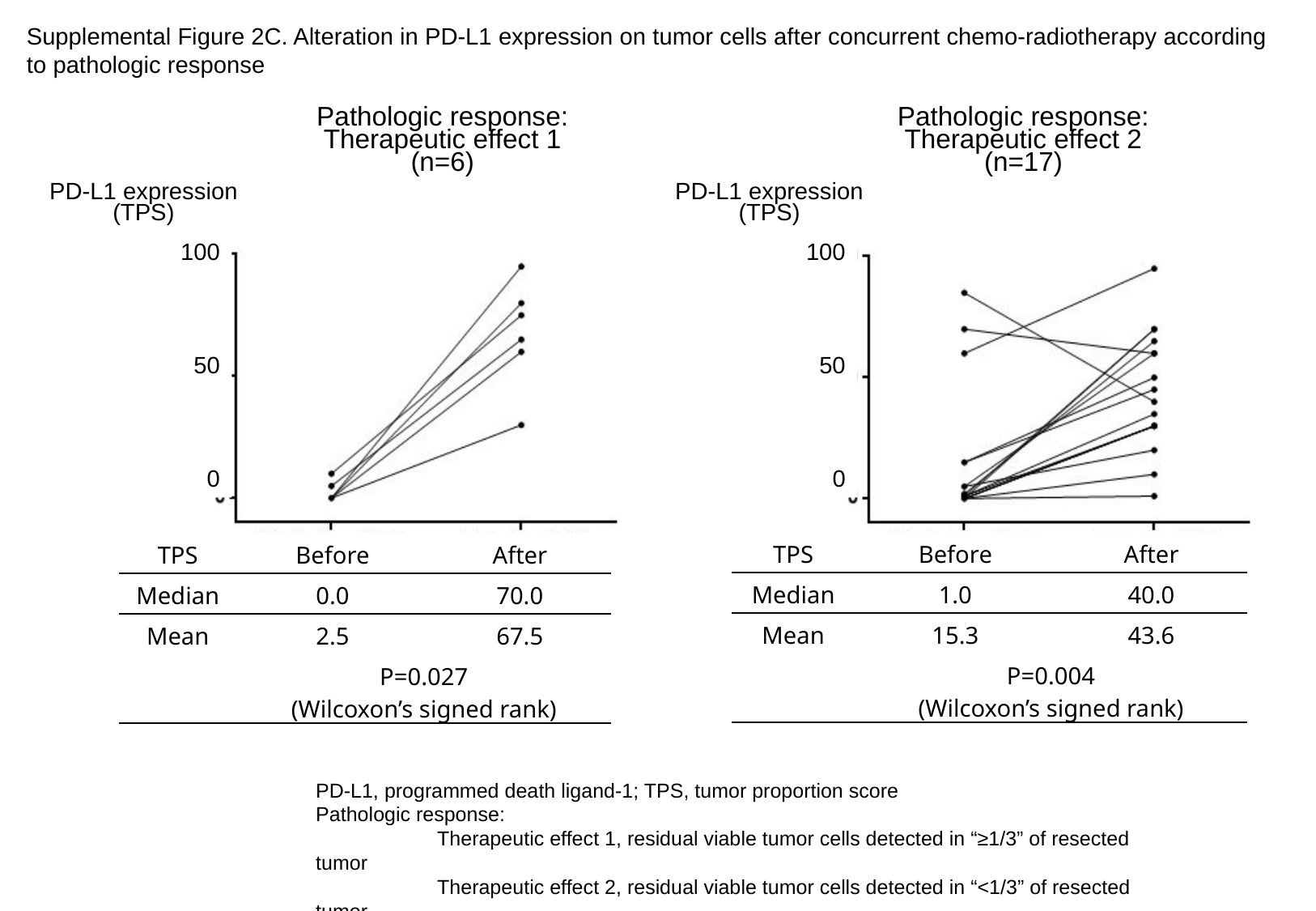

Supplemental Figure 2C. Alteration in PD-L1 expression on tumor cells after concurrent chemo-radiotherapy according to pathologic response
Pathologic response:
Therapeutic effect 1
(n=6)
Pathologic response:
Therapeutic effect 2
(n=17)
PD-L1 expression
(TPS)
100
50
0
PD-L1 expression
(TPS)
100
50
0
| TPS | Before | After |
| --- | --- | --- |
| Median | 1.0 | 40.0 |
| Mean | 15.3 | 43.6 |
| | P=0.004 (Wilcoxon’s signed rank) | |
| TPS | Before | After |
| --- | --- | --- |
| Median | 0.0 | 70.0 |
| Mean | 2.5 | 67.5 |
| | P=0.027 (Wilcoxon’s signed rank) | |
PD-L1, programmed death ligand-1; TPS, tumor proportion score
Pathologic response:
	Therapeutic effect 1, residual viable tumor cells detected in “≥1/3” of resected tumor
	Therapeutic effect 2, residual viable tumor cells detected in “<1/3” of resected tumor

## Slide 5
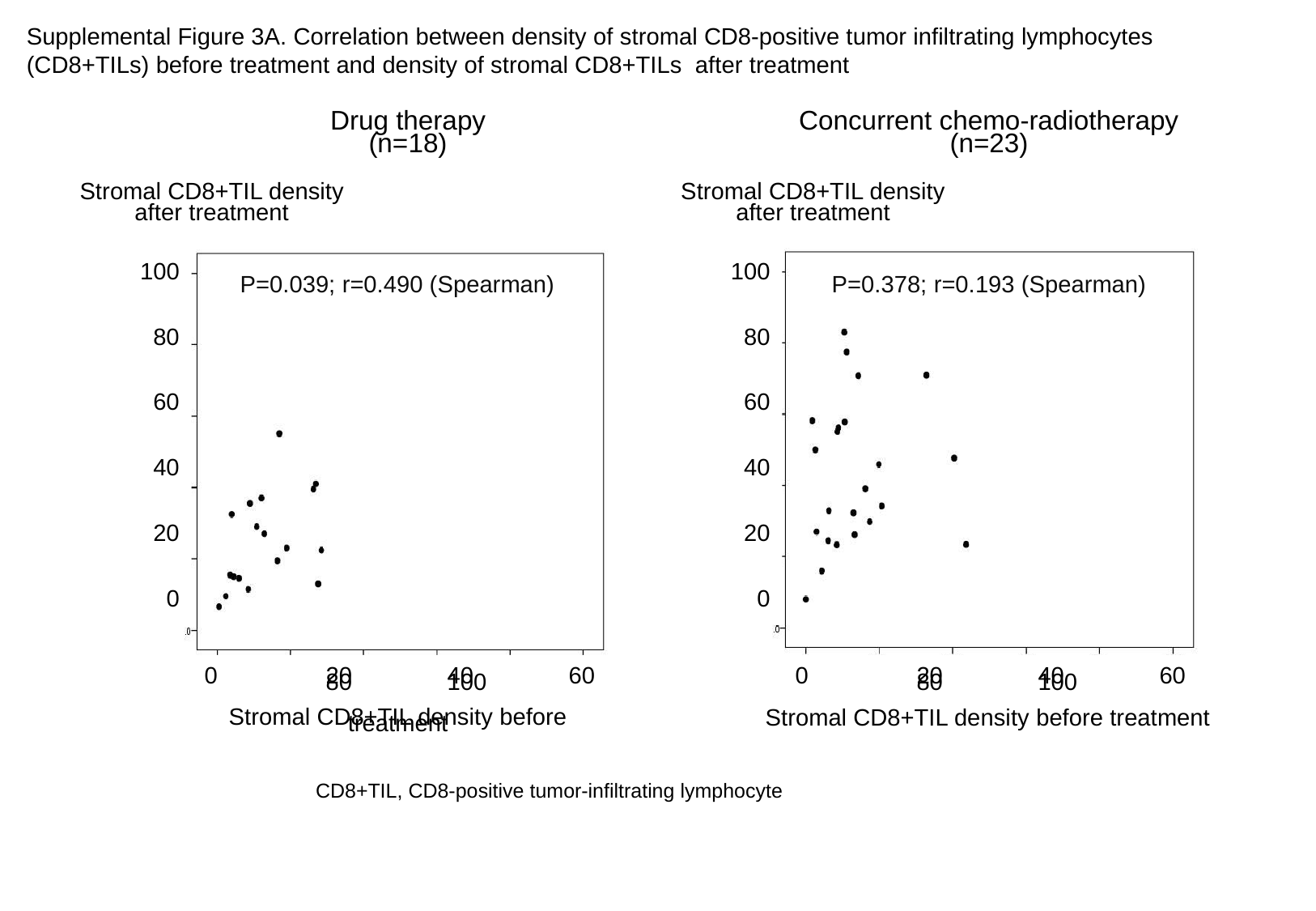

Supplemental Figure 3A. Correlation between density of stromal CD8-positive tumor infiltrating lymphocytes (CD8+TILs) before treatment and density of stromal CD8+TILs after treatment
Drug therapy
(n=18)
Concurrent chemo-radiotherapy
(n=23)
Stromal CD8+TIL density
after treatment
100
80
60
40
20
0
0	20	40	60	80	100
Stromal CD8+TIL density before treatment
Stromal CD8+TIL density
after treatment
100
80
60
40
20
0
0	20	40	60	80	100
Stromal CD8+TIL density before treatment
P=0.039; r=0.490 (Spearman)
P=0.378; r=0.193 (Spearman)
CD8+TIL, CD8-positive tumor-infiltrating lymphocyte

## Slide 6
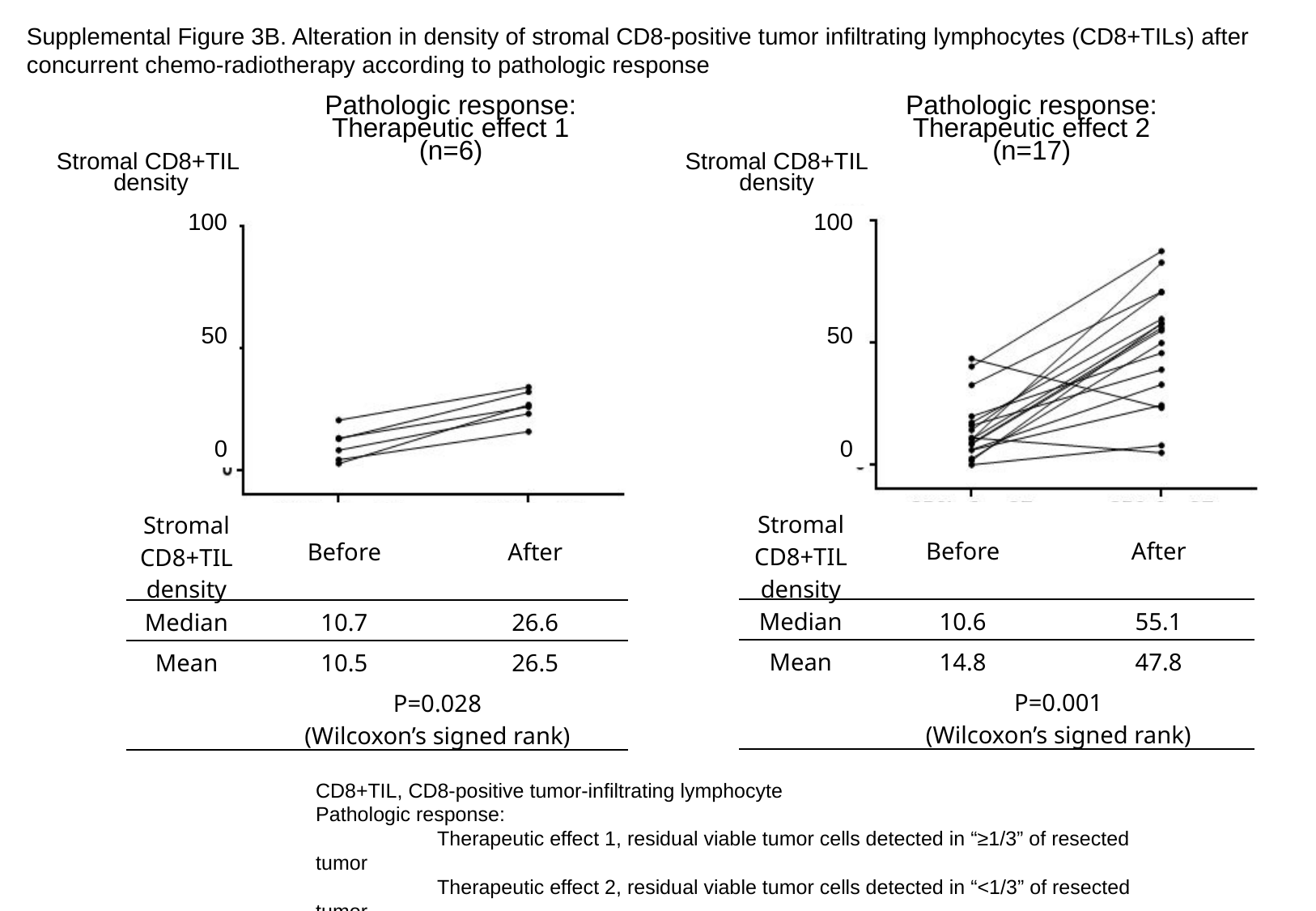

Supplemental Figure 3B. Alteration in density of stromal CD8-positive tumor infiltrating lymphocytes (CD8+TILs) after concurrent chemo-radiotherapy according to pathologic response
Pathologic response:
Therapeutic effect 1
(n=6)
Pathologic response:
Therapeutic effect 2
(n=17)
Stromal CD8+TIL
density
100
50
0
Stromal CD8+TIL
density
100
50
0
| Stromal CD8+TIL density | Before | After |
| --- | --- | --- |
| Median | 10.6 | 55.1 |
| Mean | 14.8 | 47.8 |
| | P=0.001 (Wilcoxon’s signed rank) | |
| Stromal CD8+TIL density | Before | After |
| --- | --- | --- |
| Median | 10.7 | 26.6 |
| Mean | 10.5 | 26.5 |
| | P=0.028 (Wilcoxon’s signed rank) | |
CD8+TIL, CD8-positive tumor-infiltrating lymphocyte
Pathologic response:
	Therapeutic effect 1, residual viable tumor cells detected in “≥1/3” of resected tumor
	Therapeutic effect 2, residual viable tumor cells detected in “<1/3” of resected tumor
